# A Mobile Web App to Improve Health Screening Uptake in Men (ScreenMen): Utility and Usability Evaluation Study

**DOI:** 10.2196/10216

**Published:** 2019-04-15

**Authors:** Chin Hai Teo, Chirk Jenn Ng, Sin Kuang Lo, Chip Dong Lim, Alan White

**Affiliations:** 1 Department of Primary Care Medicine University of Malaya eHealth Initiative Faculty of Medicine Kuala Lumpur Malaysia; 2 Institute for Health & Wellbeing Leeds Beckett University Leeds United Kingdom

**Keywords:** internet, mHealth, eHealth, mass screening, health behavior, men’s health

## Abstract

**Background:**

Globally, the uptake of health screening is suboptimal, especially in men and those of younger age. In view of the increasing internet access and mobile phone ownership, ScreenMen, a mobile Web app, was developed to improve health screening uptake in men.

**Objective:**

This study aimed to evaluate the utility and usability of ScreenMen.

**Methods:**

This study used both qualitative and quantitative methods. Healthy men working in a banking institution were recruited to participate in this study. They were purposively sampled according to job position, age, education level, and screening status. Men were asked to use ScreenMen independently while the screen activities were being recorded. Once completed, retrospective think aloud with playback was conducted with men to obtain their feedback. They were asked to answer the System Usability Scale (SUS). Intention to undergo screening pre- and postintervention was also measured. Qualitative data were analyzed using a framework approach followed by thematic analysis. For quantitative data, the mean SUS score was calculated and change in intention to screening was analyzed using McNemar test.

**Results:**

In total, 24 men participated in this study. On the basis of the qualitative data, men found ScreenMen useful as they could learn more about their health risks and screening. They found ScreenMen convenient to use, which might trigger men to undergo screening. In terms of usability, men thought that ScreenMen was user-friendly and easy to understand. The key revision done on utility was the addition of a reminder function, whereas for usability, the revisions done were in terms of attracting and gaining users’ trust, improving learnability, and making ScreenMen usable to all types of users. To attract men to use it, ScreenMen was introduced to users in terms of *improving health* instead of *going for screening*. Another important revision made was emphasizing the screening tests the users do not need, instead of just informing them about the screening tests they need. A *Quick Assessment Mode* was also added for users with limited attention span. The quantitative data showed that 8 out of 23 men (35%) planned to attend screening earlier than intended after using the ScreenMen. Furthermore, 4 out of 12 (33%) men who were in the precontemplation stage changed to either contemplation or preparation stage after using ScreenMen with *P*=.13. In terms of usability, the mean SUS score of 76.4 (SD 7.72) indicated that ScreenMen had good usability.

**Conclusions:**

This study showed that ScreenMen was acceptable to men in terms of its utility and usability. The preliminary data suggested that ScreenMen might increase men’s intention to undergo screening. This paper also presented key lessons learned from the beta testing, which is useful for public health experts and researchers when developing a user-centered mobile Web app.

## Introduction

### Background

In the past decade, many Web-based interventions have been developed to improve health outcomes of the public. Web-based interventions not only have a wider reach but also are less labor intensive and less resource exhaustive as compared with conventional health interventions; in addition, they can be interactive, personalized, and fun, which makes learning more effective and ultimately leads to improved users’ health behavior. In addition, the impact of Web-based health interventions is further amplified with the flux of mobile technology into health care. The mobile phone, which is a good platform to deliver health care to people anytime and anywhere, is widely available and affordable these days, including in developing countries [1]. Many studies have shown that Web-based interventions, including mobile Web apps, are effective in improving health outcomes such as improving physical activity level, asthma treatment knowledge, psoriasis knowledge, and weight loss, as well as reducing depression symptoms and preventing low back pain [2-10].

Web-based interventions could be deployed to improve health screening uptake as well. Health screening plays an important role to detect and treat diseases at an early stage, which leads to reduced mortality rate [11]. Despite its benefits, the uptake of health screening remains suboptimal, especially in men and those of younger age [12-14]. This pattern has also been observed in Malaysia as reported in the National Health and Morbidity Survey (NHMS), where only 34.9% of Malaysian men attended health screening in 2011 [15]. The NHMS also reported that the prevalence of undiagnosed hypertension was higher than known hypertension in men younger than 55 years [16]. In Malaysia, health screening can be conducted at a public hospital, public health clinic, private hospital, private clinic, as well as blood test lab. There is a public health care facility within every 5-km radius, including in the rural areas. Although the fee for utilizing a public outpatient clinic, including health screening is as low as Malaysian Ringgit (MYR) 1 (approximately US$ 0.25), health screening uptake remains low. There are many contributing factors for this trend; however, lack of awareness and knowledge on health screening could be one of the key factors [17,18].

Many types of interventions to improve the uptake of health screening in men, including those using partners’ involvement, educational workshops, reminder phone calls, and letters have been evaluated [19]. However, only educational interventions were found to be effective in improving men’s intention to undergo screening and increasing the actual screening uptake; others were inconclusive because of poor study design in terms of blinding of participant and allocation concealment [19]. There are also many Web-based interventions on health screening in men that have been evaluated, such as Web-based patient decision aid on prostate cancer screening, as well as educational Web and social media to encourage HIV screening in men [20-22]. However, these interventions are disease-specific, and there is a lack of intervention that promotes comprehensive (all in one) health screening in men, which is crucial in ensuring holistic care for men [19]. Among the recommended health screening for men by the United States Preventive Services Task Force (USPSTF) are hypertension, diabetes, dyslipidemia, colorectal cancer, lung cancer, HIV, hepatitis, sexually transmitted infections, depression, as well as lifestyle risk factors including smoking status, alcohol usage, obesity, diet, and physical activity [23]. These recommendations should be applied on the basis of men’s health profile such as age, ethnicity, and family history.

In view of the increasing internet access and mobile phone ownership in Malaysia, as well as in the world [1], ScreenMen, a mobile Web app, which aims to promote comprehensive health screening in men was developed. ScreenMen is mobile-responsive and aimed to be disseminated via mobile phone to all Malaysian men. It was developed on the basis of theories, evidence, as well as the needs of users [17-19,24]. ScreenMen was developed focusing on men in view of the lower level of health outcomes and behavior in men, as well as answering the call to use a gender sensitive approach in health intervention [25,26]. Before the development of ScreenMen, a need assessment was conducted with working men from a banking institution in Kuala Lumpur to identify their needs on health screening and to find out what men want in a health screening mobile app [18,24]. At the time of development, the prototype of ScreenMen was tested with experts from various backgrounds (alpha testing) and was revised iteratively to improve it.

Before ScreenMen was finalized, a beta testing was conducted. Beta testing aims to test a software with end users in a real-world setting to identify and rectify any potential issue before being released. This is particularly important for a mobile Web app as Web-based technologies are growing and changing rapidly [27]. Poor usability is often reported as one of the main reasons why users stopped using a mobile Web app, as a consequence of inadequate user testing [28]. To ensure that a mobile Web app is useful, experts recommend that it should be evaluated in terms of its utility (whether a website provides the features the users need), as well as usability (how easy and pleasant the features are) with users [29,30].

### Objectives

Thus, this study aimed to evaluate ScreenMen with men from the community in terms of its utility and usability, as well as to present the key revisions made to improve the utility and usability in ScreenMen, on the basis of the feedback obtained.

## Methods

### Study Design Overview

This study used a mixed-methods design to evaluate the utility and usability of ScreenMen with end users. The mixed-methods approach helps to triangulate the findings using qualitative and quantitative methods. The qualitative assessment was done using the retrospective think-aloud method with the aid of a topic guide, which contained questions on utility and usability [31,32]. Instead of the prospective think-aloud method, the retrospective think-aloud method was chosen to simulate the actual usage and to resolve any unforeseen navigability issues at users’ real-life settings [30]. A questionnaire was also used to score and evaluate ScreenMen in terms of utility and usability quantitatively. This study was approved by the University of Malaya Medical Centre Medical Research Ethics Committee (MRECID No. 201610144372).

### Study Setting, Sampling, and Recruitment

This study was conducted with healthy men from a banking institution in Kuala Lumpur, the capital of Malaysia. Unlike alpha testing with experts, which was done at the developer’s site, beta testing is conducted at the users’ settings, which was men’s working place in this study. Men from a banking institution were chosen as they were reported to have increased work-related stress, which affected their psychological as well as physical health, including smoking and alcohol overuse, depression, body posture issue, and visual problem [33,34]. Their busy schedule and excessive work demand also contributed to nonattendance of health screening [35,36]. These men represent a group of *hard-to-reach* men in the community, who often do not seek health care services despite having easy access to them.

The same banking institution where the needs assessment was conducted in the earlier phase was selected as the recruitment site for this beta testing. This study was approved by the banking institution. Men who have a mobile phone and were from the banking institution were recruited to participate in the beta testing. They were purposively sampled according to their job position, age, education level, and screening status to achieve maximal variation of the feedback on ScreenMen. A Microsoft Excel spreadsheet that contained the participants’ demography was used to plan the recruitment to ensure equal representation from each sampling criterion. Men who participated in the needs assessment phase were first contacted and arranged for interviews. Then, the snowballing method was used to recruit new participants, where the recruited participants were asked to recommend their colleagues to participate in the study. New participants were also included in addition to those who had participated in the needs assessment phase to gather more feedback on ScreenMen. All participants were reimbursed with MYR 50 (approximately US $ 12) for their time participating in this study, which took about an hour.

The sample size of a usability study is often small. Studies have shown that the optimum sample size to detect sufficient usability problem is 10 users [37]. As this study involved quantitative evaluation, at least 20 participants were aimed to be recruited to obtain statistically significant number [38]. The recruitment was stopped once data saturation was reached.

### The ScreenMen Web App (Beta Testing Version)

ScreenMen is a mobile-responsive Web app aimed to be disseminated via smartphone. It aims to educate men, empower men, and improve men’s behavior on health screening. ScreenMen was developed to contain male-sensitive attributes (such as using car maintenance analogy), as well as evidence-based recommendation for health screening gathered from the USPSTF [23]. Apart from that, 4 key sections of ScreenMen were developed following a framework modified from the health literacy principle, to guide the learning process in ScreenMen [39]. The 4 sections are as follows:

- Learn: This section contains a short educational video to demystify the misconceptions on health screening, which were identified in the needs assessment.
- Assess: This is an interactive section where users can interact with ScreenMen to assess their health risks and obtain personalized health advice, as well as the evidence-based health screening they need, on the basis of their health profile. There were 15 health conditions that were being assessed in ScreenMen, and they include obesity, unhealthy diet, physical activity, tobacco use, alcohol misuse, high blood pressure, diabetes, dyslipidemia, colorectal cancer, lung cancer, HIV, syphilis, hepatitis B, hepatitis C, and depression. This section is algorithm-driven and attempts to mimic a real-life clinical consultation with a doctor. A health report can be generated at the end of this section.
- Ask: In this section, there is a list of frequently asked questions about screening, which men can read if they would like to have further clarifications about screening. The questions were developed on the basis of the comprehensive framework on barriers and facilitators to health screening in men [17].
- Prepare: This section aims to prepare the users to undergo health screening by providing basic logistic information such as where to screen, when to screen, and cost of screening.

### Data Collection

In-depth interviews (IDIs) and focus group discussions (FGDs) were conducted for data collection. At the time of the appointment, the researchers first briefed the participants about the study using a participant information sheet. The participants were encouraged to ask questions and were informed that they could stop the study at any time. Once agreed to participate, the participants were asked to sign a consent form and fill up the demography form, including intention to undergo screening. Then, the participants were given a smartphone with ScreenMen activated on the screen. They were asked to use it themselves and notify the researchers once they had finished using it. All on-screen activities were being recorded using a free screen recording app (AZ Screen Recorder by Hecorat). The researchers were present in the same room to observe the participants’ behavior when using ScreenMen and take field notes, as well as assist the users, only when necessary.

**Table 1:** Postintervention questionnaire.

No.	Item	Strongly agree		Agree	Neither agree nor disagree	Disagree	Strongly disagree
1	I think that I would like to use this website frequently	—^a^		—	—	—	—
2	I found the website unnecessarily complex	—		—	—	—	—
3	I thought the website was easy to use	—		—	—	—	—
4	I think that I would need the support of a technical person to be able to use this website	—		—	—	—	—
5	I found the various functions in this website well integrated	—		—	—	—	—
6	I thought there was too much inconsistency in this website	—		—	—	—	—
7	I would imagine that most people would learn to use this website very quickly	—		—	—	—	—
8	I found the website very cumbersome to use	—		—	—	—	—
9	I felt very confident using the website	—		—	—	—	—
10	I needed to learn a lot of things before I could get going with this website	—		—	—	—	—
11	Does the website help you to understand more about your health risks?	—	Yes				
		—	No				
12	Does the website help you to understand more about health screening?	—	Yes				
		—	No				
13	Do you intend to go for health screening in the future?	—	Yes, in the next 1 month				
		—	Yes, in the next 6 months				
		—	Yes, in the next 1 year				
		—	Yes, in the next 2 years				
		—	Yes, in the next 5 years				
		—	No, I do not intend to go for health screening				
14	Would you recommend this website to your family or friends?	—	Yes				
		—	No				

^a^Indicate boxes for participant’s response.

Once completed, the participants were asked to answer the postintervention questionnaire, which contains the validated 10-question System Usability Scale (SUS) [40] and 4 utility questions, including intention to undergo screening (Table 1). The scale of question 13, *Do you intend to go for health screening in the future?* was developed on the basis of the transtheoretical model of health behavior change [41]. This model explained the stages of behavior change in a person, from precontemplation (no intention of change), contemplation (intent to change in the next 6 months), preparation (intent to change in the next 1 month), action (acted and maintained behavior within 6 months), to maintenance (maintained behavior more than 6 months). Only precontemplation, contemplation, and preparation were adopted for the scale of question 13 as these are related to intention to change.

Then, retrospective think aloud with playback was conducted. Using a topic guide, the researchers started the interview by asking the participants to provide their overall opinion on the Web app; to comment on its contents and layout (usability); to explain how, if the Web app helped them to understand more about health and screening; and to suggest any other part of the Web app that can be improved. The on-screen recording was played to assist the participant in the retrospective think-aloud process. They were probed to comment on the content and layout when going through each section of ScreenMen. All conversations during the retrospective think aloud were audio-recorded. For FGDs, all procedures were similar to those of IDIs, except that only 1 participant was given the project’s mobile phone, whereas others used their own mobile phones and during the feedback session, and the ScreenMen Web app was projected on the screen and navigated by the researcher, page by page, to assist the retrospective think aloud, instead of playing the on-screen recording of each participant.

### Data Analysis

The analysis was performed after completing all data collection. Subsequently, the researchers met to discuss the issues and proposed the revisions to be done on ScreenMen. The qualitative data obtained were analyzed using a framework approach to systematically guide the revision of the Web app. After each interview or discussion session, the researchers discussed and compiled a list of comments and issues on ScreenMen, with the aid of the field notes taken. The researchers then listened to the audio recording to triangulate and check for additional comments. Unlike the usual approach in a qualitative study, the audio-recordings were not transcribed verbatim in this study as the purpose of this beta testing was to capture the users’ feedback on the Web app rather than to provide an in-depth understanding of the users’ experience. The list of comments compiled was then coded under utility or usability and by section (Multimedia Appendix 1), which were then used to revise ScreenMen. In addition, to present the data in a more meaningful way in this paper, the comments and issues identified were grouped and categorized according to common themes. This was done by the first author and discussed and agreed by all authors.

For the quantitative data, all data were managed and analyzed using the IBM SPSS Statistics version 21. First, the SUS score from each participant and a mean SUS score for all participants were calculated. The SUS score was interpreted using the adjective rating scale developed by Bangor [42]. The utility questions were analyzed using descriptive statistics (percentage of yes). For intention to undergo screening, the percentages of participants who plan to screen earlier than intended, later than intended, and no change in intention after using ScreenMen, were calculated by comparing the intention to screen pre- and postintervention. Intention to screen was also analyzed according to stage of behavior change, specifically by comparing the number of participants in the precontemplation stage (more than 6 months) with the number of participants in either contemplation or preparation stage (6 months or less) after using ScreenMen, using the McNemar test.

## Results

### Participant Demography

In total, 24 men participated in the beta testing with 14 IDIs and 2 FGDs (5 participants in each FGD). They were conducted from February to March 2017. The details of the participants are shown in Table 2.

### Qualitative Evaluation (Observation and Retrospective Think Aloud)

#### Utility

The participants found the ScreenMen useful as they could learn more about their health risks, what screening to go for, and what they could do to improve their health. The same was found for older men who had undergone screening, as ScreenMen contains information they never knew before, such as colorectal cancer and the unnecessary screening tests:

I like all of it, it tells you your health, everything about where you are (in terms of health), and what you should do to improve it.

40-59 years, Senior manager

Using this web, people know what diseases they should check

40-59 years, Clerk

Now I understand about the importance of health screening. We don’t know that our lifestyle could actually affect our health. Using this website, you know what to be improved upon.

20-29 years, Sales advisor

Some mentioned that they were glad to learn about the unnecessary screening tests. One participant suggested to highlight this more to ensure all users get it:

My key take home message from this website is some of the tests are unnecessary, for example, the liver or kidney test, as it may over or under detect the disease. Nowadays, there are a lot of external blood test centers, they normally package ECG, heart stress test, and everything together and sell you thousands of Ringgit (Malaysian currency). I didn’t know that those are actually unnecessary. So, this is something I got to know now. It’s good to know that those are actually not useful for screening. This information is a little secluded and need to be highlighted better.

20-29 years, Officer

A participant was glad as:

...it contains localized contents for us (Malaysian), unlike the UK or US websites.

20-29 years, Officer

One participant mentioned that using ScreenMen may trigger men to take care of their health:

ScreenMen is easy to use, can add more knowledge and act as a trigger to take care of health when going through the website, unlike those who do not receive anything and do not do anything about health.

30-39 years, Clerk

Men also felt that ScreenMen was convenient to use.

We have limited time for screening. With this, we can check at anywhere, we can have the information and what can we do (to improve health). It is just like talking to a doctor or consultant.

30-39 years, Clerk

It is good for people. People are always with handphone. With application like this, one doesn’t need to go anywhere, at home also can do, at office also can do.

30-39 years, Clerk

will share this website with friends via Facebook Group.

40-59 years, Clerk

On the other hand, 1 participant raised the issue that users may not use ScreenMen again after using it once:

It’s good. Will I use the website? Yes. But subsequently will I continue to use it repeatedly again, it remains a question mark.

30-39 years, Senior manager

The participants also suggested that a reminder function may be useful as users may not act instantly after using ScreenMen. Thus, the research team added a function where users can input their email, and an event entitled *My Check-up Day* would appear in their email calendar, calculated on the basis of their past screening date. It serves as a reminder for users as they check their calendar daily and come across that added event.

Some participants suggested that it would be good to have a list of screening centers with phone numbers to make appointments on the website, as that may facilitate users to take action to screen. However, the research teams decided not to include the list to avoid being perceived as using it for commercial reason.

**Table 2:** Characteristics of participants in the beta testing (N=24).

Characteristics		Statistics
**Age range (years), n (%)**		
	20-29	7 (29)
	30-39	10 (42)
	40-59	7 (29)
Age (years), mean (range)		37 (23-56)
**Ethnicity, n (%)**		
	Malay	10 (42)
	Chinese	10 (42)
	Indian	3 (13)
	Others	1 (4)
**Position, n (%)**		
	Senior manager	7 (29)
	Officer	5 (21)
	Sales advisor	5 (21)
	Clerk	7 (29)
**Education, n (%)**		
	Secondary school	5 (21)
	Certificate/diploma	4 (17)
	Degree	13 (54)
	Postgraduate	2 (8)
**Marital status, n (%)**		
	Unmarried	10 (42)
	Married	14 (58)
**Screened in the past 1 year, n (%)**		
	Yes	9 (38)
**Mobile phone operating system, n (%)**		
	iOS	11 (46)
	Android	12 (50)
	Windows	1 (4)
**Participated in needs assessment, n (%)**		
	Yes	13 (54)

#### Usability

Overall, participants mentioned:

[ScreenMen] is quite user-friendly, comfortable to look at, and not too cluttered. The interface is easy to understand, not too complicated.

20-29 years, Officer

The participants also felt assured to use the Web app as it was stated upfront that the Web app does not capture any identifiable information from them.

There were several key issues with revisions to improve ScreenMen, and they were grouped under 3 themes: (1) attracting and engaging users, (2) ensuring effective learning, and (3) catering for the widest range of users’ characteristics.

##### Theme 1: Attracting and Engaging Users

###### Designing a Simple and Focused Home Page

**Figure 1:**
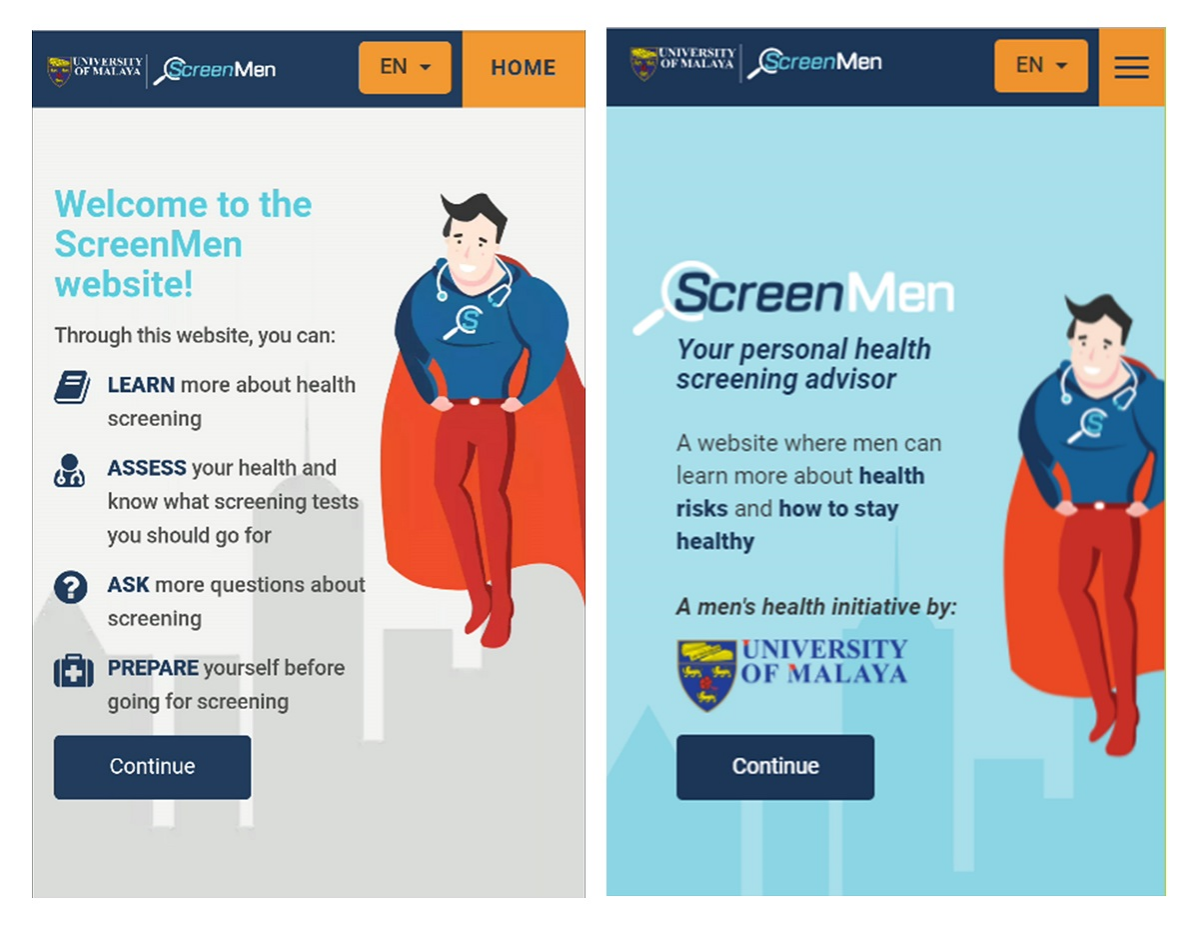
The home page of ScreenMen before and after revision.

The home page of ScreenMen outlined the 4 key sections of the Web app. Some participants found that the home page contained too much information to read and felt that it may put off users. Thus, the home page was simplified to include only the main objective of what users may gain from this Web app (Figure 1).

###### Promoting the Concept of Health Instead of Just Screening

Men were less interested in screening as they did not understand about screening and its importance. Describing the Web app as a platform to learn about screening did not interest the users. However, men wanted to be healthy, and they more readily received information that can keep them healthy. Thus, the objective on the home page was framed in terms of learning about users’ health risks and ways to stay healthy, instead of learning about health screening. Additional health information, such as erectile dysfunction and urinary symptoms, were also added as requested by men, to provide more information than just screening.

###### Highlighting the Credibility of the Web App

The participants felt that there was a lack of credibility on the home page. They mentioned that the credibility of Web app is crucial to gain users’ trust so that they continued to use the Web app. To address this issue, they suggested to enlarge the university’s logo on the home page.

###### Incorporating a Male-Favored Avatar

The ScreenMen Web app attempted to attract men using Dr ScreenMen, a Superman-resembling doctor avatar at the home page. Although some liked Dr ScreenMen figure as it encourages them to be strong, especially those from lower educational level, others had no comment on the Dr ScreenMen figure. One participant suggested to make Dr ScreenMen provide various types of reaction, but this was not done because of technical complexity and the potential impact on Web loading time.

**Figure 2:**
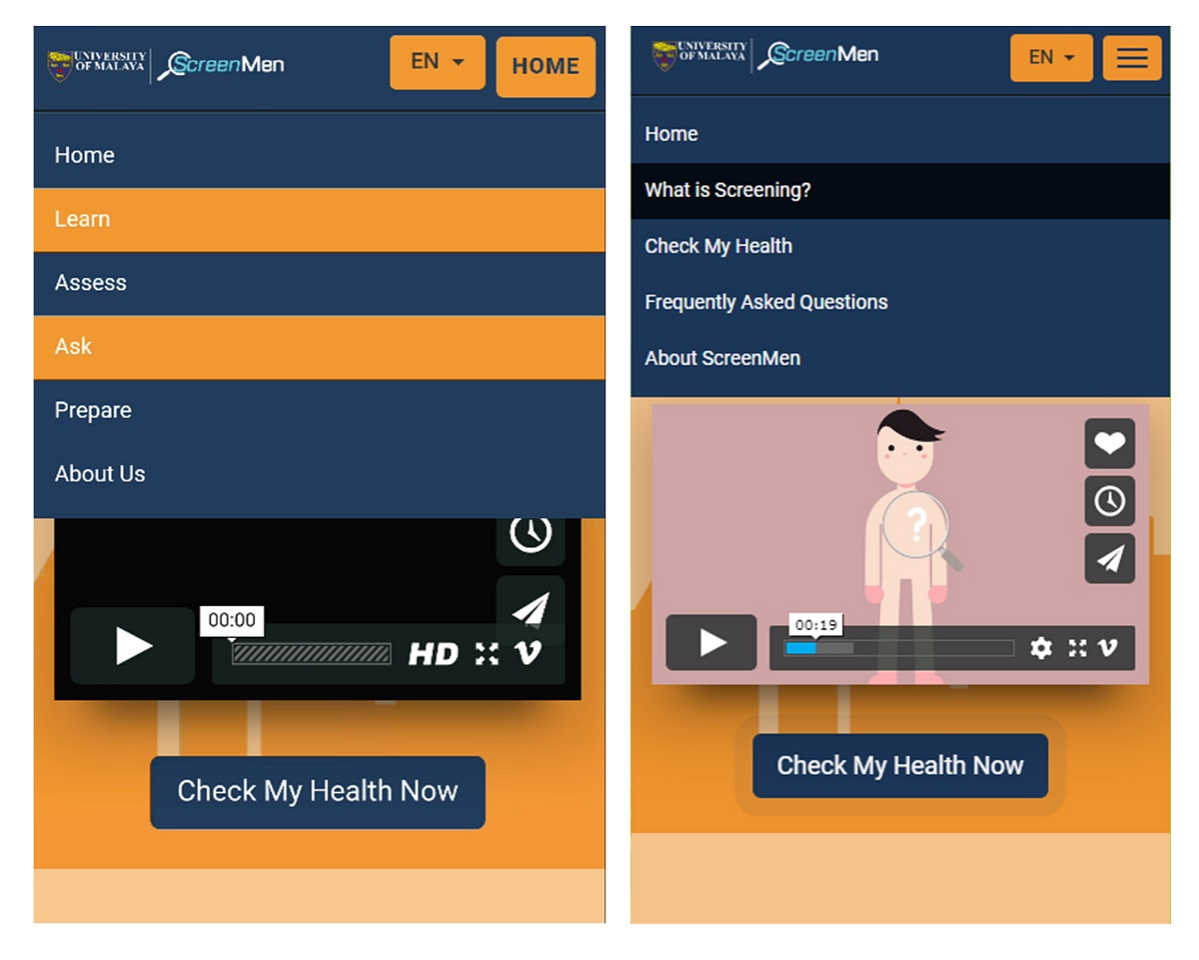
The menus of ScreenMen before and after revision.

##### Theme 2: Ensuring Effective Learning

###### Using Practical Terms Instead of Theoretical Concepts

From the researchers’ observation, the Learn, Assess, Ask, and Prepare menus were unclear, and participants were confused about these concepts. For example, some thought that they could ask questions to a doctor at the *Ask* section and were lost looking for that function. Theoretical concepts were difficult to be understood by users, and thus, the menus *Learn*, *Assess*, and *Ask* were revised to *What is Screening?*, *Check My Health*, and *Frequently Asked Questions*, to more accurately represent the content of each Web app section (Figure 2). The *Prepare* section was removed and merged into *Check My Health*.

###### Using Linear Learning Design for a More Structured Learning

Users are allowed to navigate freely to any section of ScreenMen by using the icons on the home page. This was done to cater to users who had already understood the basics of health screening and the repeat users. However, some users were confused as they went to the third section directly from the home page and did not go through the first and second section. Thus, the navigation links on the home page were removed. Users who liked to skip any section could use the *hamburger* button.

###### Incorporating Concepts That Are Familiar to Men

Most participants agreed that the car maintenance analogy was very useful in helping them learn about health screening. The only comment on this was to use the word *car service* instead of *car maintenance*, as the term is more commonly used among men. However, this change was not made as *maintenance* is closer to health screening concept, where maintenance is about the routine schedule for service, whereas service is about the task performed on a vehicle.

###### Showing Important Information First Instead of Optional Information

At the time of the usability testing, it was found that some users lost their attention at the third section (Ask). The *Ask* section contains a long list of frequently asked questions, and most users only skimmed through them. The fourth section contains a small amount of information to prepare users for screening, which is crucial for them to learn. To ensure that users learn this crucial information before losing attention, they were brought forward and merged into the last part of the second *Check My Health* section. The *Frequently Asked Questions* section was made optional as most information in this section was presented in the earlier sections.

###### Emphasizing the “Negatives” When Addressing Misconceptions

**Figure 3:**
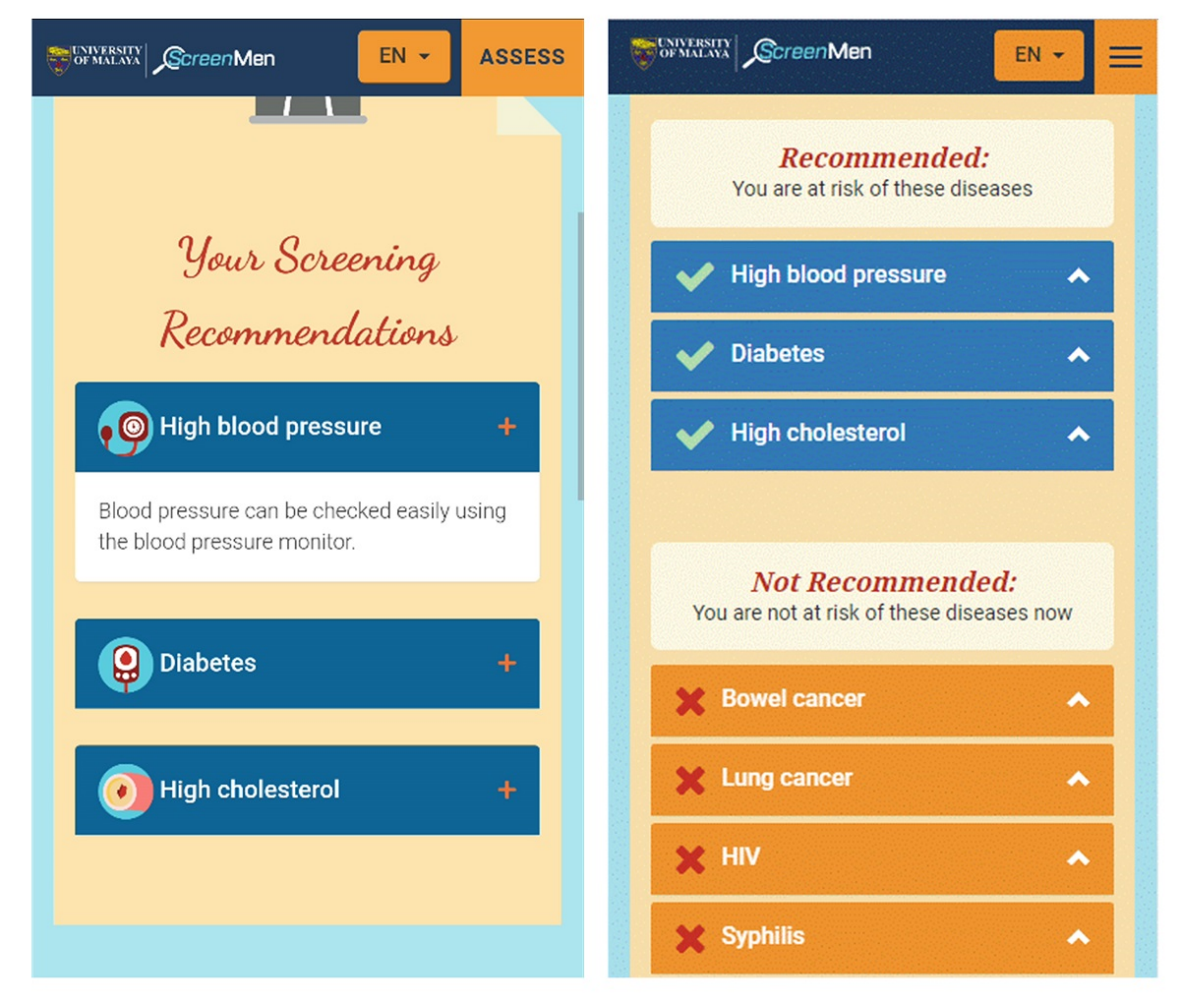
User’s list of screening recommendation without and with emphasis of not recommended screening.

**Figure 4:**
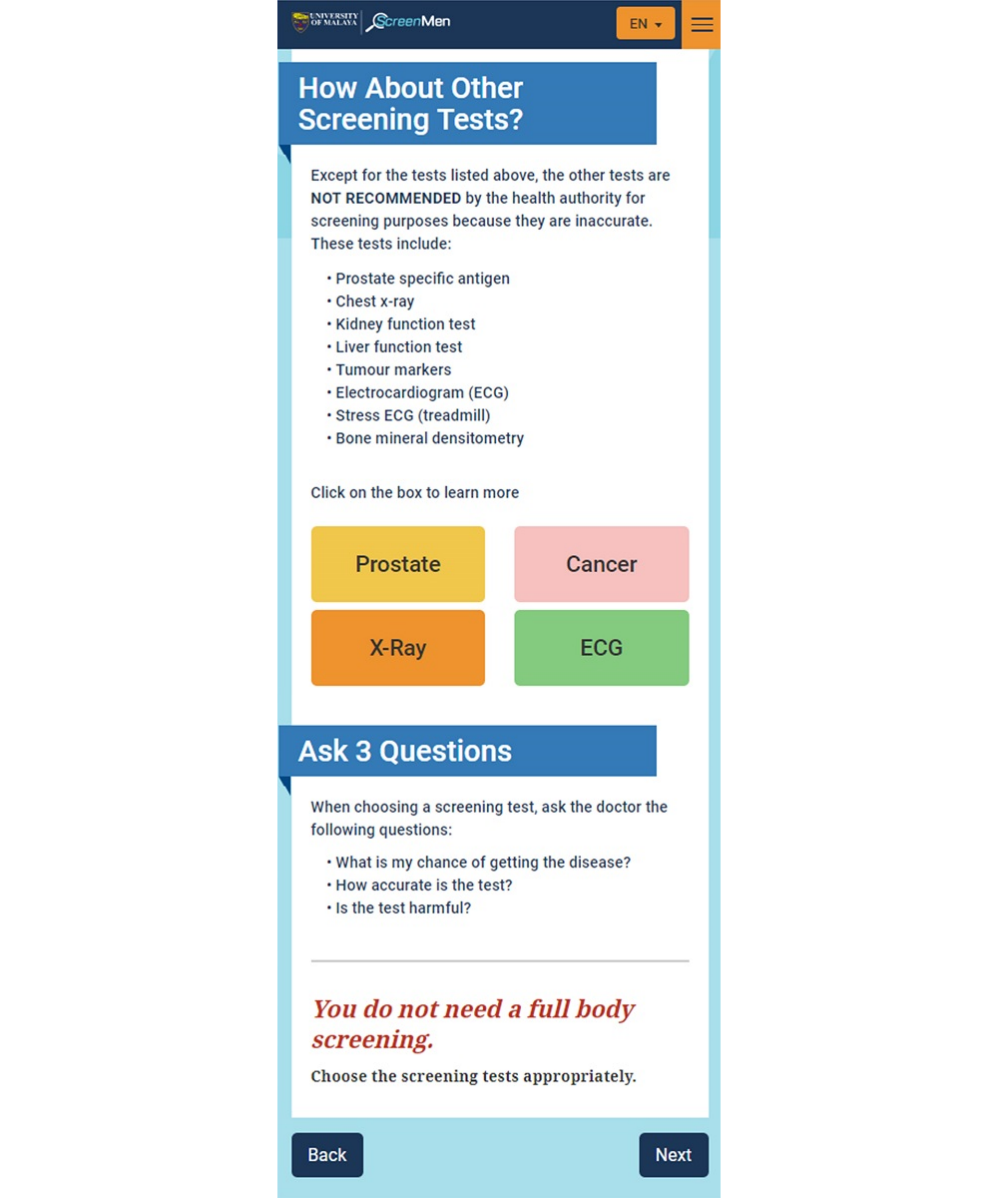
The newly added section to encourage users to avoid unnecessary screening.

ScreenMen was developed on the basis of the USPSTF guidelines. It advocates evidence-based screening and encourages users to avoid unnecessary screening. To fulfill the *personalized content* factor as suggested by the users during needs assessment, ScreenMen only states the screening tests users need to undergo on the basis of their health profile. However, after using the Web app, it was found from the retrospective think aloud that the users still had the mindset of *undergoing more screening tests or full body screening is better*. It is insufficient to inform men only on the screening tests they need, but it is also necessary to emphasize the screening tests they do not need, especially when addressing misconceptions. To more effectively educate men to avoid unnecessary health screening, ScreenMen was revised to emphasize the tests that they do not need to go for (Figure 3). Some of the unnecessary screening tests, which were commonly done in the community, were highlighted with reasons why they should be avoided (Figure 4). ScreenMen also empowers men to avoid unnecessary screening by encouraging them to ask the doctors 3 questions when choosing screening tests.

##### Theme 3: Catering for the Widest Range of Users’ Characteristics

###### Suiting Lower Literacy Users

Some participants commented that there was too much information to be read in ScreenMen, especially for people with a lower literacy level. The information in ScreenMen was thus simplified to present only relevant and brief information. Links for additional information were incorporated throughout the Web app for users who may want more information about certain topics.

###### Anticipating the Lowest Level of Users’ Health Behavior

Developed from the medical perspective, users had problems answering some of the questions in ScreenMen. For example, some participants had difficulties answering *I take fruit __ time(s) a day*, as they do not eat fruit every day. This question was developed partly for the recommendation of *5 servings of fruits and vegetables per day*. As a result of the problem, this question was revised to *weekly* instead of *daily*, as weekly fruit intake was more prevalent among users. The algorithm was also revised to recalculate the users’ input to compare against the recommended level.

Another example was the blood pressure reading. Most users could not remember their blood pressure reading but remembered that their blood pressure was normal from the previous health screening. To provide a more accurate advice, an option of *I don’t know but I know my BP is normal* was added instead of letting these users select *I don’t know*.

###### Providing a Quick Mode Option for Busy Men

The health assessment section was developed to mimic a real-life clinical consultation with a doctor. This section starts with Dr ScreenMen greeting the users, obtaining users’ age, and followed by health assessment, topic by topic. Dr ScreenMen asks question and provides advice on each health condition on the basis of users’ answers. At the end of this section, users can view the summary of their health status with screening recommendation.

**Figure 5:**
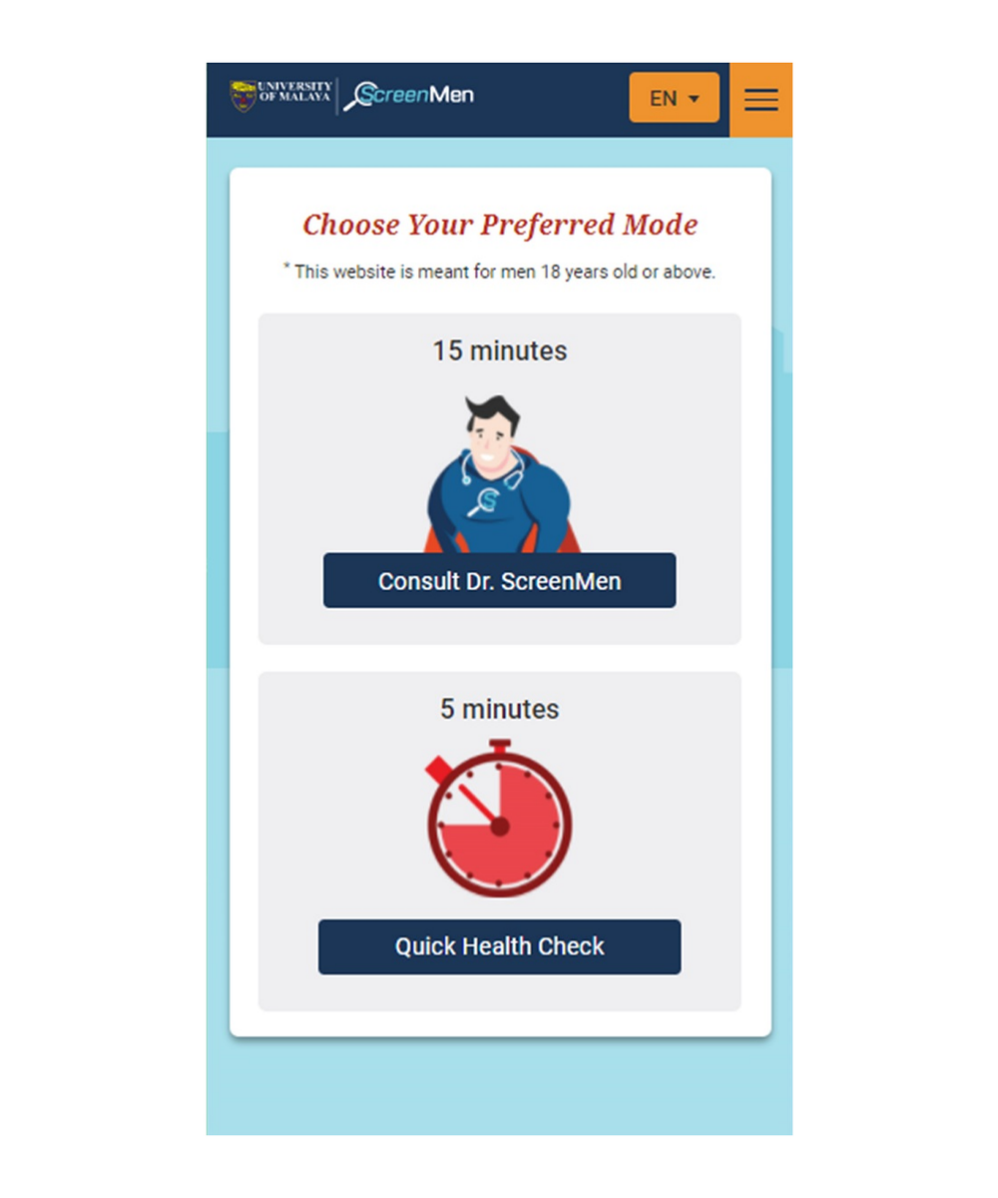
Options of consultation or quick mode for health assessment.

However, some participants commented that men who are busy may not like to go through this process and would prefer a shorter mode. Though the consultation mode is more ideal for learning as it breaks the session into chunks, a *Quick Health Check* mode was added as an alternative to cater to *busy users* (Figure 5).

###### Accommodating Female Users

Though ScreenMen was developed for men, some participants suggested that it could also be used by women as a woman might be the person taking care of her husband or father in a family. Some of the sentences were thus rephrased to accommodate female users; for example, *only men 18 years old or above should use this website* to *this website is meant for men 18 years old and above*.

###### Taking Into Account the Difference in Culture

Malaysia consists of 3 main ethnic groups: Malay, Chinese, and Indian. There were only 2 languages available in ScreenMen for beta testing (English and Malay). Some Chinese participants mentioned that their parents may need the Mandarin version as they were not literate in English and Malay languages. The Mandarin language was thus added to ScreenMen. No issue was raised regarding having Tamil language on ScreenMen as Indians are usually literate in English and Malay.

Apart from language, some sections of ScreenMen might be sensitive to certain ethnic groups. For example, all users were assessed in terms of alcohol intake, which may not be relevant to Muslim users as alcohol intake is prohibited in the religion. However, the Muslim participants reassured the research team that it was not an issue as the option *I never drink alcohol* was already in place.

Another concern was the sexually transmitted disease assessment. Personal information such as having multiple sexual partners, having sex with men, and injecting drugs was being asked of the users. However, the participants mentioned that they had no hindrance in answering these as no identifiable information was being recorded, and these were important for them to know.

### Quantitative Evaluation (Questionnaire)

Only 23 participants answered the postintervention questionnaire as 1 participant was called for work urgently. The details of the postintervention quantitative evaluation are shown in Table 3. The SUS score obtained (mean 76.4, SD 7.72) indicated that the ScreenMen had good usability (good usability score range: 71.4-85.5) [42]. All participants agreed that they understood more about their health risks and health screening after using ScreenMen, and would recommend it to others.

For intention to undergo screening, 8 out of 23 men (35%) planned to attend screening earlier than intended after using the ScreenMen (no intention to 2 years, n=1; 5 years to 1 year, n=1; 2 years to 1 month, n=1; 1 year to 6 months, n=3; and 6 months to 1 month, n=2); 14 out of 23 men (61%) did not change; whereas 1 out of 23 men (4%) planned to screen later (1 month to 6 months). In terms of stage of behavior change, 4 out of 12 (33%) men, who were in precontemplation stage, changed to either contemplation or preparation stage after using ScreenMen (Table 4). However, the change from precontemplation (more than 6 months) to either preparation or contemplation stage (6 months or less) after using ScreenMen was not statistically significant different as McNemar test revealed *P*=.13. Furthermore, 11 out of 23 men were already in contemplation/preparation stage before using ScreenMen.

**Table 3:** Quantitative evaluation after using the ScreenMen (N=23).

Postintervention evaluation		Statistics
System Usability Scale score, mean (SD)		76.4 (7.72)
Understand more about their health risks, n (%)		23 (100)
Understand more about health screening, n (%)		23 (100)
Will recommend ScreenMen to others, n (%)		23 (100)
**Change in the intended time to screen (before and after using ScreenMen), n (%)**		
Earlier		8 (35)
No change		14 (61)
Later		1 (4)

**Table 4:** Intention to screen by stage of behavior change before and after using ScreenMen (N=23).

Stage of behavior change	Preintervention, n (%)	Postintervention, n (%)
Precontemplation (>6 months)	12 (52)	8 (35)
Preparation/contemplation (≤6 months)	11 (48)	15 (65)
Total	23 (100)	23 (100)

## Discussion

### Principal Findings

This study found that ScreenMen is acceptable to men in terms of its utility and usability. The participants found ScreenMen useful as they could learn more about their health risks and the evidence-based health screening they should go for. They also felt that ScreenMen was convenient to be used and may trigger men to undergo screening. The quantitative data showed that many men planned to undergo screening earlier than initially intended after using ScreenMen, although there was no significant difference when analyzed on the basis of the stage of behavior change using McNemar test. The participants also felt that ScreenMen was user-friendly and comfortable to look at. The SUS score also indicated that ScreenMen had good usability.

Tackling the issue of health screening is not an easy endeavor, and ScreenMen had to be further improved in terms of utility and usability. The key improvement in terms of utility was the addition of a reminder function. Past studies have shown that reminder interventions including those using a letter, email, and short message service were effective in increasing screening uptake [43,44]. ScreenMen also had to be framed in terms of *improving health* instead of *going for screening*, as men generally do not see the importance of health screening but would like to know more about staying healthy. As a result of the lack of interest in screening, ScreenMen had to be shortened and simplified to ensure that key messages were delivered in the shortest possible time. The most challenging part was advocating evidence-based health screening. Additional efforts and emphases needed to be placed for men to internalize the message to avoid unnecessary health screening. These were crucial for policy makers and researchers to consider when developing interventions in the future, particularly on topics that are surrounded by misconceptions and have low public interest.

This study has taken a male-sensitive approach to improve men’s behavior. ScreenMen was developed specifically for men and incorporated with male-familiar contents such as the car maintenance concept and the *Superman-like* Dr ScreenMen figure. The car maintenance concept has been used in several programs globally to encourage health screening such as the Man MOT, which is a suite of Web-based health information and advice services, where men can chat with a National Health Services General Practitioner service anonymously on any health topic [45]. MOT stands for the Ministry of Transport, which was the responsible Ministry for the road worthiness test in the United Kingdom. It was used to name this program because of men’s familiarity with it. Men reported that they felt empowered using it and were likely to use it again, especially as the first port of call for nonemergency health issues [45]. In addition, the use of [26,46-49] Dr ScreenMen avatar might be useful too, as there are preliminary studies that showed promising results of using avatars or embodied conversational agents (ECA) in health interventions, where an ECA is capable in engaging and motivating users in terms of learning and behavioral change [50-52].

The outcomes of this utility and usability testing may appear differently if ScreenMen was developed as a mobile app instead of a mobile Web app. The reminder function would be easily built, and more interesting functions such as alert, monitoring function, daily health messages, and integration with social media can be included. However, the research team decided to develop ScreenMen in the form of mobile Web app for the ease of dissemination. Though this hindered having more useful functions in ScreenMen, reaching out to men is seen as a more important factor, as a health screening mobile Web app or mobile app is not something being sought after by men as they do not see the importance of health screening, unlike for exercise or diet apps. A Web app has a broader dissemination than an app as it can be accessed instantly without needing to download and install, can be shared quickly among friends, and can be viewed on a computer as well [53]. This factor is important to be considered by public health experts and researchers, especially when addressing health issues that are not seen to be important by the public.

The findings from this beta testing reinforced the importance of conducting testing with end users. Though many iterations of testing were done with experts during alpha testing, some of the issues were not captured. For example, the fruit intake per day question was not seen as a problem to experts but posed difficulties for users to answer. Other than that, the experts felt that the amount of text was just right; however, it was still too much for some participants. Apart from testing with experts, the development team has also considered many usability guidelines such as the International Organization for Standardization and the International Electrotechnical Commission (ISO/IEC) 25010 software product quality model and Nielsen usability diagram [29,54]. Nevertheless, many usability issues still emerged. The nuance of usability issues would only emerge during the in-depth beta testing with end users.

### Strengths and Limitations

There are several strengths and limitations in this study. The strength of this study was that we managed to sample men from a wide demographic range, which gave rise to maximal variations of the qualitative findings. The multifaceted approach used (quantitative and qualitative, observation and retrospective think aloud with playback, IDI and FGD) allowed the study to gather a rich amount of data and enabled data triangulation. With regard to limitations, the design of the utility section in the questionnaire limited the data analysis. The questions on *understand more about health risks*, *understand more about health screening*, and *recommend this website to family and friends* should provide a Likert scale instead of *Yes* and *No* to enable a more meaningful analysis. For the intention to screen question, instead of fixing the options on the basis of the stage of behavior change, an open-ended field that allows participants to enter their actual number of months to screen would also allow better analysis. The sample size for quantitative analysis, though sufficient for the SUS as recommended by experts, was inadequate for the utility questions, especially the McNemar test for intention to screen. In addition, because of purposive sampling reason, about half of the participants were already in the contemplation or preparation stage even before using ScreenMen, which further diminished the analyzable sample size. Nevertheless, this study’s primary focus was on the qualitative findings, which aimed to identify issues so that ScreenMen could be improved. The quantitative data were just the preliminary effectiveness findings, which will be measured more definitively in a trial. For the qualitative method, although ScreenMen was meant to be tested in a *real-world* setting, the researcher was present in the same room to observe and to assist the users, in case any technical issue occurred. This may affect how the users used ScreenMen, as they might feel being monitored and obliged to use ScreenMen properly, unlike at home. However, this gave more gain than loss, as observation on users’ behavior provides very important data for probing during interview; nevertheless, solving technical issues is also important to prevent errors in the future.

### Conclusions

This study showed that ScreenMen is acceptable to men in terms of its utility and usability. Men are able to learn more about their health risks and screening via ScreenMen. The preliminary data suggested that ScreenMen might increase men’s intention to undergo screening and may potentially improve the actual uptake of health screening as well. Further evaluation in the form of randomized controlled trial should be conducted to determine the effectiveness of ScreenMen in improving the uptake of evidence-based health screening. Apart from that, this study also allowed further refinement of ScreenMen to improve its utility and usability. We have shared the key lessons learned from this beta testing, which might be useful for public health experts and researchers to develop user-centered mobile Web apps in the future.

## Appendix

Multimedia Appendix 1 The framework used for data analysis.

## Abbreviations

ECA: embodied conversational agent
FGD: focus group discussion
IDI: in-depth interview
MYR: Malaysian Ringgit
NHMS: National Health and Morbidity Survey
SUS: System Usability Scale
USPSTF: United States Preventive Services Task Force
