# Supplementary material for: A Mobile Web App to Improve Health Screening Uptake in Men (ScreenMen): Utility and Usability Evaluation Study
Source: JMIR Mhealth Uhealth. 2019 Apr 15;7(4):e10216. doi: 10.2196/10216 (PMC6487344; doi:10.2196/10216)
Supplement: Multimedia Appendix 1 [file mhealth_v7i4e10216_app1.docx]

Appendix 1. The framework used for data analysis.

| **Section** | | **Utility** | | **Usability** | |
| --- | --- | --- | --- | --- | --- |
|  |  | **Comment** | **Solution** | **Comment** | **Solution** |
| Home | Participant |  |  |  |  |
|  | Observer |  |  |  |  |
| Learn | Participant |  |  |  |  |
|  | Observer |  |  |  |  |
| Assess | Participant |  |  |  |  |
|  | Observer |  |  |  |  |
| Ask | Participant |  |  |  |  |
|  | Observer |  |  |  |  |
| Prepare | Participant |  |  |  |  |
|  | Observer |  |  |  |  |
| About us | Participant |  |  |  |  |
|  | Observer |  |  |  |  |
